# Soaring energetics and glide performance in a moving atmosphere

**DOI:** 10.1098/rstb.2015.0398

**Published:** 2016-09-26

**Authors:** Graham K. Taylor, Kate V. Reynolds, Adrian L. R. Thomas

**Affiliations:** Department of Zoology, University of Oxford, Oxford OX1 3PS, UK

**Keywords:** dynamic soaring, static soaring, flight performance, wind drift, gliding, wing morphing

## Abstract

Here, we analyse the energetics, performance and optimization of flight in a moving atmosphere. We begin by deriving a succinct expression describing all of the mechanical energy flows associated with gliding, dynamic soaring and thermal soaring, which we use to explore the optimization of gliding in an arbitrary wind. We use this optimization to revisit the classical theory of the glide polar, which we expand upon in two significant ways. First, we compare the predictions of the glide polar for different species under the various published models. Second, we derive a glide optimization chart that maps every combination of headwind and updraft speed to the unique combination of airspeed and inertial sink rate at which the aerodynamic cost of transport is expected to be minimized. With these theoretical tools in hand, we test their predictions using empirical data collected from a captive steppe eagle (*Aquila nipalensis*) carrying an inertial measurement unit, global positioning system, barometer and pitot tube. We show that the bird adjusts airspeed in relation to headwind speed as expected if it were seeking to minimize its aerodynamic cost of transport, but find only weak evidence to suggest that it adjusts airspeed similarly in response to updrafts during straight and interthermal glides.

This article is part of the themed issue ‘Moving in a moving medium: new perspectives on flight’.

## 1. Introduction

The moving atmosphere presents opportunities and challenges alike for the animals that fly through it. Opportunities for energy harvesting are afforded by the updrafts that facilitate static soaring, and by the spatio-temporally varying wind fields that facilitate dynamic soaring. Yet, each opportunity presents its own challenges. Air that is rising in one place must be replaced by air that is sinking in another, so unfavourable downdrafts are always present in the vicinity of an updraft. Likewise, the turbulence associated with a varying wind field presents obvious challenges to flight stability [1]. Even a constant wind field will affect the optimization of heading and airspeed, which presents further challenges for sensing the wind, and adjusting velocity appropriately in response [2–6]. None of these themes is new, but we look at them here with fresh eyes, through the lens of some new theoretical development, and with the aid of empirical data collected using state-of-the-art onboard instrumentation.

Whereas the theory of static soaring is simple, the theory of dynamic soaring is not. Most theoretical developments of dynamic soaring [7–14] have involved modelling detailed flight trajectories, which requires the use of equations of motion whose complexity obscures the underlying physics [15,16]. Our approach here is different, and follows the guiding principle advocated by Taylor & Thomas [17], which is to make the model as general as possible, so as to identify the key physical constraints within which natural selection operates. We achieve this by framing our theoretical analysis of soaring flight in terms of its energetics, rather than in terms of its dynamics (see also [18,19]). We then use this energetic analysis to inform an empirical analysis of airspeed optimization in the gliding flight of a soaring bird.

Most studies of how birds respond to a moving atmosphere are necessarily field-based. Previous studies have measured flight trajectories from the ground using optical techniques or radar tracking [20–22], or have estimated airspeed and sink rate from a glider accompanying the bird [23]. More recently, it has become possible to measure fine-scale flight trajectories and detailed flight performance using onboard instrumentation carried by the bird. Most such studies have used either GPS units [24–30] or accelerometers [28,29], which measure movement in an Earth-fixed or inertial frame of reference. However, the miniaturization of such technologies has now reached the point that a large bird can carry all of the same instrumentation as an unmanned aircraft, including a full inertial measurement unit (IMU) and a pitot tube for measuring airspeed [1,31]. This means that onboard instrumentation can now be used to estimate how a bird is moving relative to the air, as well as the ground. Our second guiding principle is therefore to build models that can be parametrized using the kind of empirical data that are now available from onboard instrumentation [1,31].

The paper is divided into two parts. In the first part (§2), we undertake a theoretical analysis of flight in a moving atmosphere, deriving an equation that expresses all of the energy flows in soaring (§2a). We next make use of this equation to analyse the aerodynamic cost of transport in a moving atmosphere, and to explore how airspeed should be adjusted with respect to wind velocity if the cost of transport is minimized (§2b). To facilitate quantitative predictions, we then elaborate upon the classical theory of the glide polar, providing a cross-species analysis that compares the predictions of the glide polar under different aerodynamic models (§2d). Finally, we offer a new presentation of the predictions of the glide polar, in the form of a glide optimization chart that can be plotted for any given species of bird (§2e). In the second part of the paper (§3), we test our theoretical predictions using empirical data collected using onboard instrumentation carried by a captive steppe eagle *Aquila nipalensis* (§3a). This allows us to test whether and how airspeed is adjusted in relation to headwind speed (§3b), updraft speed (§3c) and the combination thereof (§3d). As these sections are quite self-contained, we conclude with only a brief discussion at the end (see §4).

## 2. Theoretical analysis of flight in a moving atmosphere

We begin by deriving a succinct expression describing all of the mechanical energy flows associated with soaring flight, which we then use to explore the optimization of gliding in an arbitrary wind field.

### (a) Energetics of soaring flight

**Figure 1.**
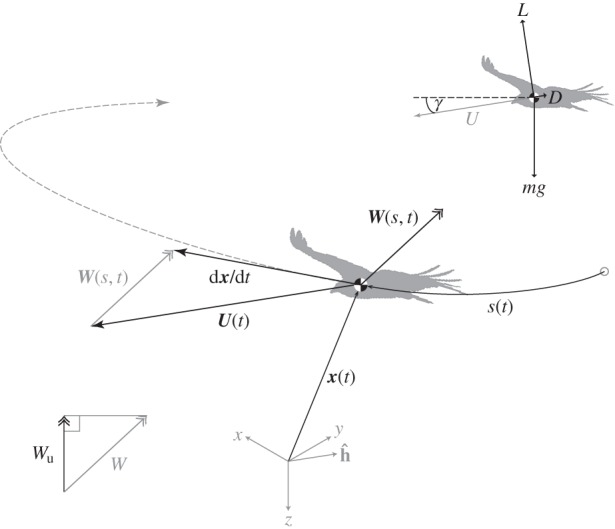
Definition sketch of the key vectors, scalars and axis systems used in the analysis of soaring energetics and gliding. Position vector (***x***) of bird in Earth-fixed coordinate system {*x*, *y*, *z*}; path coordinate (***s***) of bird; time (*t*). Local wind velocity (***W***), relative to ground. Wind speed (*W*); updraft speed (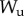). Air velocity of bird (***U***), relative to ground. Headwind direction vector (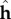). Lift (*L*), drag (*D*), weight (*mg*). Glide angle relative to air (*γ*). See text for formal definitions.

Energy conservation laws hold in an inertial frame of reference only, but mechanical energy itself can be defined in either an inertial or a non-inertial frame. The aerodynamically useful mechanical energy of a bird (*E*), defined as the mechanical energy available to do work on the air, is the sum of the bird's potential energy relative to the Earth and its kinetic energy relative to the air [32]. We may write this as
2.1
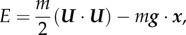
where *m* is the bird's mass, 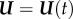 is its air velocity vector, 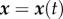 is its inertial position vector and ***g*** is the gravitational acceleration vector. Each of these vectors is assumed to be resolved in some non-rotating Cartesian axis system (figure 1). Differentiating with respect to time (*t*) gives the rate of change in the useful mechanical energy as
2.2
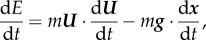
which is also the quantity measured by the total energy variometer in a sailplane [33]. The time derivative of the bird's inertial position is the vector sum of the bird's velocity relative to the air, and the air's velocity relative to the ground, so we may write the identity 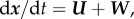 where 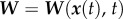 is the local wind velocity (figure 1). Differentiating with respect to time and rearranging yields the new identity 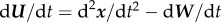. Substituting both identities into equation (2.2) and rearranging, we have
2.3
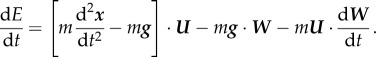
Equation (2.3) captures all of the mechanical energy flows associated with soaring flight, and it can be rewritten using only scalar quantities by making use of the fact that the scalar product of two vectors is equal to the magnitude of one vector multiplied by the magnitude of the other's projection onto it.

By Newton's second law, the square brackets in equation (2.3) comprise the total force acting on the bird (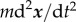) minus its body weight (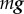). This is equal to the total aerodynamic force, of which only the component acting in the direction of the bird's air velocity (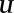) can contribute to the first scalar product. As this component is equal to the net forwards aerodynamic force of thrust 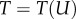 minus drag 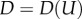, we may rewrite the first scalar product as 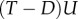, where *U* is the bird's airspeed. This term measures the rate at which the net forwards aerodynamic force does work on the air, and thereby represents the net aerodynamic power surplus or deficit. It follows that a gliding bird, with 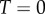, loses useful mechanical energy at a rate 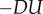, through a loss of either airspeed or altitude. These losses can be offset by converting chemical energy into mechanical energy through flapping, or by harvesting mechanical energy from the atmosphere through the soaring mechanisms represented by the other scalar products in equation (2.3).

The second scalar product in equation (2.3) (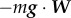) is equal to the bird's scalar body weight (*mg*) multiplied by the wind's vertical updraft component (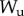). This term, which we may rewrite as 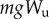, represents static soaring and predicts the rate of energy gain in both thermal and orographic updrafts alike. It measures the rate at which gravitational potential energy is harvested when flying in an updraft (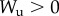), or lost when flying in a downdraft (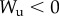). This rate is independent of the details of the bird's flight, depending only upon the strength of the updraft and the weight of the bird, so it is possible for a bird to gain energy through soaring in both gliding and flapping flight. The common kestrel (*Falco tinnunculus*) is an obvious example of the latter, routinely hover-soaring in weak updrafts. However, there are many other soaring species, such as the common crane (*Grus grus*) [34], European bee-eater (*Merops apiaster*) [35], and lesser kestrel (*Falco naumanni*) [30], that habitually mix flapping and gliding in weaker thermal conditions. This presumably allows them to exploit updrafts that would be unsuitable for sustained soaring on fixed wings.

The third scalar product in equation (2.3) (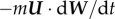) is equal to *mU* multiplied by the scalar projection of the time derivative of the wind (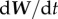) onto the headwind direction vector (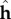), defined as the unit vector opposite to the bird's air velocity vector (figure 1). We will write this projection as 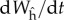, and can therefore rewrite the third scalar product as 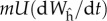. It should be noted that the projection of the time derivative of the wind onto 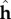 is *not* identical to the time derivative of the projection of the wind onto 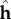, which reflects the fact that a bird cannot harvest aerodynamically useful mechanical energy from a constant wind field merely by turning into the wind. The resulting term 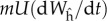 represents dynamic soaring, and measures the rate at which useful kinetic energy is obtained from a time-varying wind field. Because 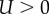, it is clear by inspection that energy will be gained through dynamic soaring if and only if 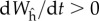, which is satisfied when flying in either a strengthening headwind or a weakening tailwind. The principle of dynamic soaring can therefore be captured in one very simple rule: *fly upwind in a strengthening wind and downwind in a weakening wind*.

It is possible to break this dynamic soaring term down further, by noting that the time derivative 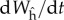 could reflect either the explicit time-dependence of the headwind in a temporally varying wind field, or the implicit time-dependence of the headwind seen as a bird progresses through a spatially varying wind field. These two cases correspond to gust soaring and shear soaring, respectively, which we can make explicit by restating the wind locally as 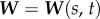, where 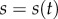 is a path coordinate describing the bird's instantaneous position along its flight trajectory relative to the ground. Differentiating ***W*** with respect to both variables, and substituting the result into equation (2.3) with the other identities above, we arrive at the expression
2.4
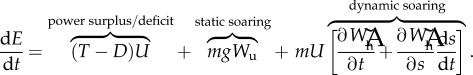
The first of the dynamic soaring terms in the square brackets represents gust soaring and measures the rate at which useful kinetic energy is harvested or lost in a time-varying wind field. Gust soaring has been discussed in the context of sea-soaring Procellariiformes such as petrels and albatrosses [36,37], but is also used by land-soaring birds that play the wind. For example, we have observed Eurasian jackdaws (*Corvus monedula*) that were gliding into a strong wind suddenly gain several metres of altitude when blown backwards by a gust, after which they would fly back downwind at tremendous groundspeed (G. K. Taylor 2015, personal observation). The second of the dynamic soaring terms in the square brackets represents shear soaring and measures the rate at which useful kinetic energy is harvested or lost in a spatially varying wind field. Shear soaring has historically been thought to be important in petrels and albatrosses flying in the wind gradient low over the oceans [32], but might also be important to land-soaring birds flying in the shear layers generated in the lee of a ridge or other obstruction. In any case, because windspeed usually increases with altitude, the general rule for dynamic soaring can be restated for shear soaring as follows: *descend downwind; ascend upwind*.

Before concluding this section, we should note that it would have been possible to undertake a complementary analysis in which kinetic energy was defined with respect to groundspeed rather than to airspeed. Such an analysis could appear to lead to different conclusions regarding the mechanism of shear soaring, because the largest gain in kinetic energy relative to the ground occurs when a bird turns downwind near the top of the shear layer [13,28], which is not associated with any gain in kinetic energy relative to the air [15,16]. However, it is the bird's airspeed—not its groundspeed—which determines the aerodynamic force that is produced. Moreover, the fundamental source of energy in shear soaring is the variation in wind speed that the bird encounters as it passes through the shear layer [32]. Hence, while it is possible to use either formulation to solve for flight trajectories that result in no net change in mechanical energy over a period of cyclical ascent and descent [28,38], the mechanism by which useful kinetic energy is gained must be understood in relation to the bird's movement relative to the air, not the ground. This accords with the original verbal description of the mechanism by Rayleigh [32].

### (b) Aerodynamic cost of transport in a moving atmosphere

Equation (2.4) is practically useful as a means of evaluating the net rate of change in the mechanical energy of a bird (or vehicle) when flying in a given wind field, and can also be used to explore the optimization of glide speed in relation to the wind (see also [2–6]). For example, equation (2.4) shows that a bird gliding in a uniform wind field expends useful mechanical energy at a rate
2.5
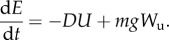


**Figure 2.**
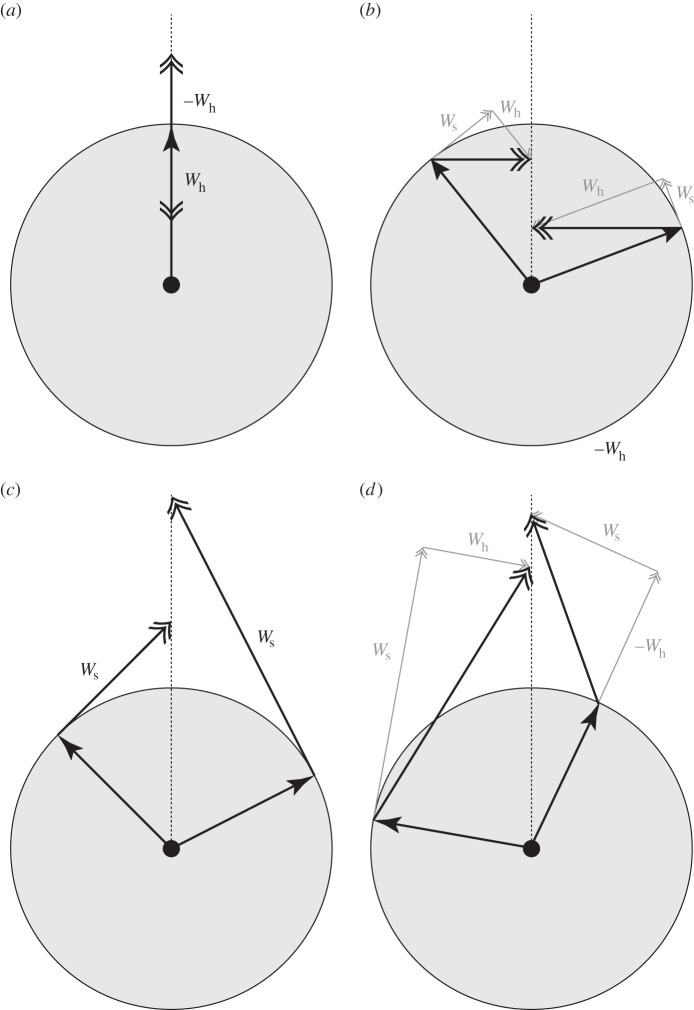
Clock diagrams of wind drift correction for a variety of different wind speeds and directions. The ‘hands’ of the clocks represent the horizontal air velocity of the bird, as a unit vector on the unit circle. The black double-headed arrows represent the horizontal wind vectors, which are resolved into their headwind (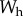) and sidewind (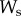) components as appropriate. The diagrams are drawn so that the resultant ground velocity is the vector (not drawn) from the centre of the unit circle to the tip of the wind vector, and the air velocity vector is directed so that this ground velocity vector always points to 12 o'clock. If the tip of the wind vector falls inside the unit circle, then the wind is detrimental and will increase the cost of transport. If the tip of the wind vector falls above the unit circle, then the wind is beneficial and will decrease the cost of transport. (*a*) Examples of a pure headwind (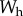) and a pure tailwind (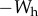). (*b*) Examples of a pure crosswind, perpendicular to the ground velocity vector (which itself runs along the dashed line). (*c*) Examples of a pure sidewind (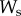), perpendicular to the air velocity vector. (*d*) Arbitrary combinations of a sidewind (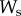) with a headwind (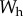) or a tailwind (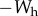).

If we assume that the bird is gliding at a shallow angle (*γ*) with respect to the air such that the horizontal component of its air velocity is 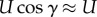 (figure 1), then it will cover ground along its flight path (*r*) at a rate
2.6
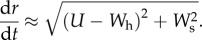
Here, the horizontal projection of the wind velocity vector (figure 2) has been resolved into its headwind (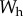) and sidewind (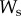) components pointing opposite to and perpendicular to the bird's air velocity vector, respectively.

These definitions of headwind and sidewind in relation to the bird's air velocity vector differ from those used in previous studies [4], which have defined headwind and crosswind in relation to the bird's ground velocity vector. The aerodynamic vector basis that we have used here is natural in the context of our analysis of soaring energetics (§2a), and would probably be natural to a bird using visual feedback from translational optic flow to maintain an intended direction of travel over the Earth. This is because the forward translational optic flow that a bird experiences depends upon the difference between the bird's airspeed and the headwind speed, whereas the lateral translational optic flow depends upon the sidewind speed. When the bird's heading is correctly adjusted, the headwind will be sensed as a retarding tendency via the mismatch between the bird's airspeed and the forward translational optic flow. Any accompanying sidewind will be sensed through the lateral translational optic flow as an assistive tendency to drift the bird back towards its desired flight track (figure 2*b*). This contrasts with the drift that a pilot attends to when lining up with a linear feature such as a runway. In this case, it makes obvious sense to resolve the wind into a headwind component parallel to the runway and a crosswind component perpendicular to it, because it is the crosswind—not the sidewind—that tends to drift the aircraft away from the line of the runway (see also figure 2*b*).

Combining equations (2.5) and (2.6) gives
2.7
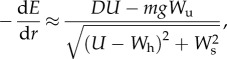
which we may interpret as the aerodynamic cost of transport in gliding flight (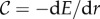), if we assume that the bird is compensating for wind drift so that it is flying along its intended track (figure 2). Obviously enough, equation (2.7) predicts that the cost of transport is decreased by flying in an updraft (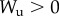) or a tailwind (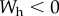), and increased by flying in a downdraft (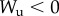) or a headwind (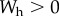). Equation (2.7) also shows that the cost of transport is decreased when flying in a pure sidewind perpendicular to the bird's air velocity (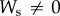, with 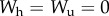). This is a special case, however, because our assumption that the bird is compensating for wind drift means that, for a given airspeed, a pure sidewind is only possible for a unique set of combinations of wind speed and direction relative to the bird's intended track (figure 2*c*). For example, a crosswind perpendicular to the bird's ground velocity will always comprise an assistive sidewind component (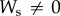) and a retarding headwind component (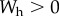) when resolved in relation to the bird's air velocity, and it is clear on geometric grounds (figure 2*b*) that their net effect will be to increase the cost of transport (see also [4]).

A gliding bird that needs to cover a set distance, or to fly as far as possible, may be expected to adjust its airspeed so as to minimize its aerodynamic cost of transport. This optimization can be explored by differentiating equation (2.7) with respect to airspeed and equating the derivative to zero, but the general solution is made cumbersome by the dependence of drag upon airspeed, and by the coupling of the headwind and sidewind components to airspeed that results from correcting for wind drift. We present the results of this optimization graphically later (figure 4), neglecting the effects of sidewinds for clarity of presentation. For now, we present only a simplified mathematical analysis, which offers analytical insights into the problem. We can simplify this analysis by noting that the cost of transport in still air is equal to the drag (see equation (2.7)), and is therefore minimized at minimum drag. At glide equilibrium, the minimum drag (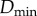) is achieved at the best glide speed (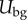). We may therefore determine qualitatively how updrafts and headwinds affect the optimization of airspeed by evaluating the airspeed derivative of the cost of transport at the point 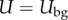. This yields
2.8
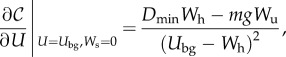
where we have set 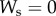 to avoid coupling the headwind component to airspeed, and where it is assumed that 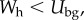 so that the bird is always making forward progress. Evidently, the sign of this derivative is determined only by the terms in its numerator, because the denominator is always positive.

It is clear by inspection of equation (2.8) that headwinds (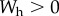) and downdrafts (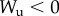) will both make the cost of transport an increasing function of airspeed for a bird that is flying at its best glide speed, so that birds should fly faster than their best glide speed under these unfavourable wind conditions. The converse applies for tailwinds (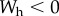) and updrafts (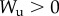), so that birds should fly slower than their best glide speed under these favourable wind conditions. Comparing terms in the numerator of equation (2.8), it is clear that the effect of a small downdraft or updraft exceeds the effect of a similar headwind or tailwind by a factor on the order of 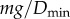, which is approximately equal to the lift-to-drag ratio in a shallow glide. Moreover, it is obvious on geometric grounds that the effect of a small downdraft or updraft will exceed the effect of a similar sidewind by an even greater degree. Other things being equal, airspeed correction ought therefore to be around an order of magnitude more important in respect of vertical as opposed to horizontal air movements—especially for birds of high glide efficiency. Of course, if the updraft exceeds the sink rate of the bird, then the aerodynamic cost of transport will be negative (see equation (2.7)), and it may no longer make sense to treat the cost of transport as a minimand at all [2,5,6].

To conclude, this entire section can be summarized in one very simple rule: *fly slower in a favourable wind and faster in an unfavourable wind*. It is worth emphasizing that we have reached this conclusion without making any assumptions about the aerodynamics, other than that drag has a minimum at some reasonable airspeed.

### (c) Classical theory of the glide polar

The theory in this section is quite classical, but we detail it here for the sake of clarity and self-containedness. The total drag that a bird experiences when gliding is a function of its airspeed, and may be represented as the sum of three distinct contributions: (i) an induced drag component resulting from the flow induced by the vortices trailing in the wake; (ii) a profile drag component resulting from friction drag, and to a lesser extent pressure drag, on the wings; and (iii) a parasite drag component resulting from pressure drag, and to a lesser extent friction drag, on the body and tail. For reasons that need not concern us here, and which are well explained elsewhere [37,39], the total drag at equilibrium is expected to be
2.9
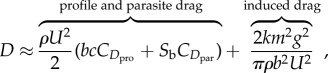
where 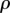 is air density, *b* is wing span, *c* is wing mean chord and 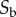 is body frontal area, and where it is assumed that the bird is gliding at a shallow angle with respect to the air, such that lift is approximately equal to body weight. Here, *k*, 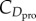 and 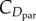 are numerical coefficients characterizing the induced drag, profile drag and parasite drag, respectively, which we will treat as constants for the time being. It is clear from equation (2.9) that whereas profile drag and parasite drag are expected to increase with airspeed, induced drag is expected to decrease with airspeed, other things being equal.

We already know that the aerodynamic cost of transport is minimized by minimizing the drag when gliding in still air (equation (2.7)). Taking the partial derivative of equation (2.9) with respect to airspeed (*U*), setting the result equal to zero to find the minimum 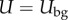, and multiplying by 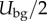, we arrive at the expression
2.10
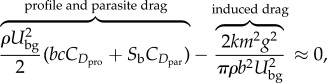
which shows that the best glide speed, 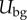, at which the cost of transport is minimized for a given flight morphology, is the airspeed at which the induced drag equals the combined parasite and profile drag. This result holds for any given flight morphology, and can therefore be adapted to analyse the effects of variable flight morphology.

Gliding birds are able to vary their planform continuously in flight, but empirical studies have shown that wing area, 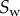, typically varies linearly with wing span [40,41]. It follows that wing mean chord (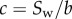) must be approximately constant, which presumably reflects the geometric constraints associated with the wing morphing mechanisms of birds. Taking the partial derivative of equation (2.9) with respect to wing span (*b*), setting the result equal to zero to find the minimum 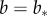 and multiplying by 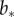, we find that
2.11
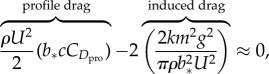
which shows that the wing span (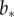) at which the aerodynamic cost of transport is minimized for a given airspeed, is the span at which the induced drag equals half the profile drag. This conflicts with the outcome of the airspeed optimization (equation (2.10)), which shows that the induced drag equals the combined parasite and profile drag if a bird is flying at the best glide speed (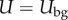) for its given span. It follows that 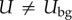 in equation (2.11), which means that when a bird is flying at the wing span 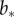 that minimizes its cost of transport for a given airspeed, it is not flying at the airspeed that would minimize the cost of transport for that wing span. This being so, we should not expect to see any change in wing span with increasing airspeed until the point at which the induced drag equals half the profile drag, which will always be at an airspeed higher than 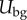. Beyond this point, wing span is expected to decrease with further increases in airspeed, so as to maintain the same ratio of induced and profile drag.

Rearranging equation (2.11), we find that the aerodynamic energetic cost of transport is minimized if wing span varies according to the following relationship (see also [37,39])
2.12
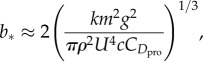
at airspeeds high enough that the induced drag would be less than half the profile drag if the wings remained fully extended. Thus, if birds adjusted their wing span so as to maximize flight efficiency, then they would be expected to start reducing span at some airspeed higher than 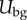 according to the proportionality 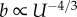. In reality, birds tend to reduce wing span over the full range of airspeeds at which they operate, and do so in an approximately linear fashion [37]. It follows that the significance of the relationship in equation (2.12) is not that it predicts how a bird typically adjusts its wing span, but rather that it allows us to place an upper limit on the glide performance that a bird can possibly achieve by its varying span. This is the best that we can do in the absence of detailed measurements of how wing span varies with airspeed in a given species, and we therefore make extensive use of equations (2.9)–(2.12) to predict flight performance throughout all the subsequent sections of the manuscript.

It now remains for us to relate the drag curve in equation (2.9) to the measurable flight performance of the bird. Equation (2.5) shows that a bird gliding in a constant wind field loses mechanical energy at a net rate 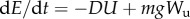. Because the kinetic energy is constant at equilibrium, it follows that this change in mechanical energy must be entirely owing to a change in the gravitational potential energy. Hence, if the bird is changing altitude at an inertial sink rate 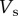 with respect to the ground, where 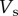 is signed negative when the bird is sinking, then its gravitational potential energy must be changing at a rate 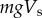. We may therefore write down the identity 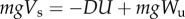 and combine this with equation (2.9) to yield the inertial glide polar
2.13
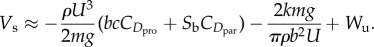
The significance of this equation is that it predicts the sink rate relative to the ground, which is what can be measured on a free-flying bird using a barometer. To arrive at the aerodynamic glide polar, which predicts the aerodynamic sink rate (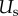), we need only subtract 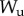 from both sides of equation (2.13).

### (d) Model uncertainty in the glide polar

In order to do anything more quantitative with the theory of the glide polar, it is necessary to know the values of its numerical coefficients. Different workers have made different assumptions, but the parasite drag coefficient (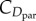) has proven especially controversial, with entire papers dedicated to its estimation. In fact, for a given species, the value of 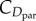 has been lowered by as much as 75% between successive versions of the most widely used aerodynamic model [37,39]. Specifically, under the current version of Pennycuick's popular Flight software [37], a value of 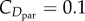 is assumed for all species, compared with values in the range 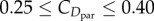 in the earlier version [39]. Science is supposed to be self-correcting, of course, and it is appropriate that assumed values be revised in the light of new experimental data, but this discrepancy emphasizes the extent of the uncertainty in 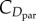, which is further compounded by the uncertainty in the body frontal area that it multiplies.

Body frontal area (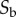) has been measured for only a handful of species, so is usually estimated from body mass, using an empirical scaling relationship 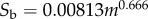 fitted to a narrow and taxonomically biased sample of 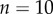 species of waterfowl and raptors [42]. The product 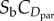 is known as the equivalent flat plate area of the body, because a flat plate with this area would produce the same drag if it blocked the flow completely. On the pragmatic grounds that neither 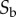 nor 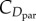 is known accurately or independently for most species, Taylor & Thomas [17] proposed letting 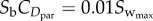, where 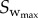 is the maximum wing area. This approximation roughly coincides with field estimates of the parasite drag on diving passerines [43], rounded down to account for the lower parasite drag expected on the streamlined bodies of larger birds. Applying this approximation to the morphological dataset assembled by Taylor & Thomas [17] produces equivalent flat plate areas that fall between the estimates given by the two versions of Pennycuick's Flight software [37,39] for 368 (82%) of the 450 species of bird. Under this approach [17], the parasite drag is assumed to be equivalent to the drag on a flat plate with 1% of the area of the wings at their maximum extent. Coincidentally, this would make the parasite drag on a 0.14 kg dollarbird *Eurystomus orientalis* equal to the flat-plate drag on a quarter dollar coin, at 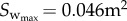 [17].

A value of 
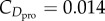
 is assumed for the profile drag coefficient in both versions of Pennycuick's model [37,39], but Taylor & Thomas [17] have proposed setting 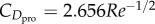, which is the classical laminar flow solution for the friction drag on a flat plate, where 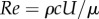 is the chord Reynolds number with 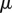 as the dynamic viscosity of the air. This has the effect of making the profile drag coefficient a function of airspeed, which will, in turn, affect the optimization of airspeed, but the variation in 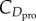 within a species is expected to be small, and can probably be safely ignored. The induced drag factor (*k*) has proven less controversial, as it is more easily modelled and less easily measured. It is generally assumed that 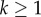 (but see [22]), with 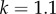 used by default in both versions of Pennycuick's model [37,39]. Taylor & Thomas [17] rounded this down to 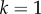, which is the value for an efficient elliptically loaded wing, but for reasons of consistency we will assume that 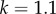 throughout, which allows for a more straightforward comparison of the effects of the uncertainty in 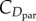 and 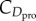.

**Figure 3.**
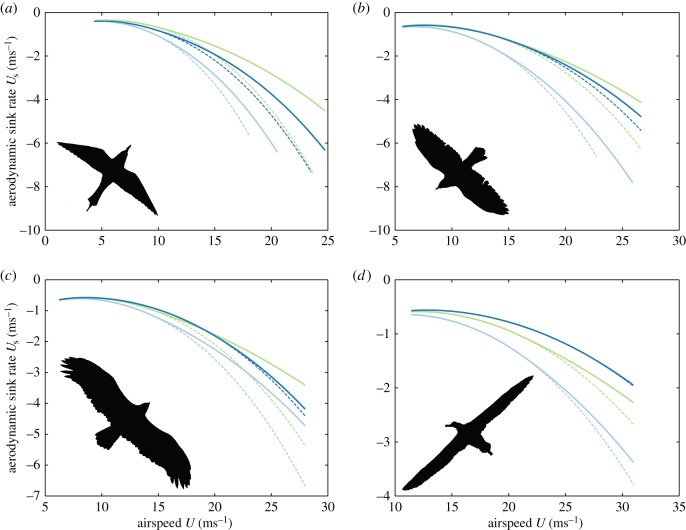
Model uncertainty in the aerodynamic glide polar. (*a*) European bee-eater (*Merops apiaster*); (*b*) Eurasian Jackdaw (*Corvus monedula*); (*c*) steppe eagle (*Aquila nipalensis*); (*d*) wandering albatross (*Diomedea exulans*). Colours denote the three published aerodynamic models discussed in the text: light blue [39], green [37], dark blue [17]. Solid lines show the best achievable glide performance given optimal span adjustment to minimize the aerodynamic cost of transport; dotted lines show performance at maximal span. The minimum airspeed plotted in each panel is the notional stall speed, assuming a maximum lift coefficient of 1.8. The maximum airspeed is arbitrary, save that the glide polars are truncated early if the predicted glide ratio falls below 3.3, which guarantees to within 5% the assumption that lift is approximately equal to body weight. See table 1 for morphological data.

In the light of this uncertainty, it is important to quantify how the predictions of the glide polar vary in relation to the assumed values of the drag coefficients. We do this by plotting the glide polar for four species of soaring bird (figure 3), for each of the three sets of parameters discussed in the preceding paragraphs [17,37,39], at both maximal and optimal span (see [46] for a formal uncertainty analysis). These four species cover most of the variation in body mass, wing loading and aspect ratio that is found in soaring birds, whereas the three models cover most of the variation in the values of the drag coefficients assumed in the published literature. It is clear that these three models show significant variation in the aerodynamic sink rate predicted for a given airspeed, especially at higher airspeeds and for larger birds. It follows that the detailed quantitative predictions of these models cannot be taken at face value without careful validation. In the following sections, we aim to provide just such a validation using data from an IMU and pitot tube worn by a captive bird in soaring flight. This will require us to develop our presentation of the glide polar one step further, which we do in §2e.

**Table 1.** Morphological measurements of four soaring birds. Maximum wing area (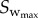) is defined as the total projected wing area, including the area of the body between the wing bases; maximum wing span (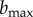) is measured from tip to tip [39]. Steppe eagle measurements are from the individual used in the flight tests.

species	scientific name	mass *m* (kg)	span 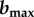 (m)	wing area 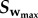 (m^2^)	source
European bee-eater	*Merops apiaster*	0.057	0.47	0.027	[44]
Eurasian jackdaw	*Corvus monedula*	0.245	0.65	0.068	[44]
steppe eagle	*Aquila nipalensis*	2.35	1.90	0.54	this paper
wandering albatross	*Diomedea exulans*	8.55	3.01	0.58	[45]

### (e) Beyond the glide polar

**Figure 4.**
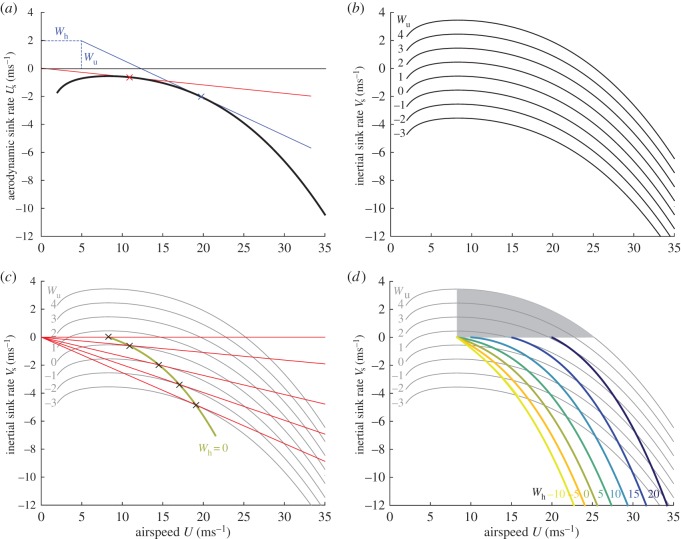
Optimization of airspeed in gliding flight. (*a*) Aerodynamic glide polar. The lines tangent to the curve identify the airspeed at which the aerodynamic cost of transport is minimized (crosses), under still conditions (red) and assuming a wind (blue) comprising a downdraft (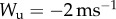) and headwind (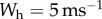). (*b*) Inertial glide polar, plotted for the different updraft speeds (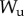) marked at the end of each curve. (*c*) Inertial glide polar, with red lines tangent to the curves identifying the airspeed at which the cost of transport is minimized under conditions of zero headwind (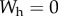), for each of the updraft speeds (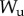). The olive line is the locus of points at which the cost of transport is minimized under these conditions. (*d*) Glide optimization chart. The grey lines denote the inertial glide polars for a range of different updraft speeds (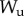). The coloured lines plot the loci of points at which the cost of transport is minimized for each of the different headwind speeds (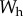). For any specific combination of headwind (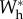) and updraft speed (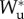), the optimum combination of airspeed and inertial sink rate is found at the intersection of the polar curve for 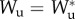 and the locus for 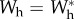. The grey shading corresponds to the region of the graph for which the updraft speed exceeds the aerodynamic sink rate, where the optimization breaks down.

We have taken a mathematical approach to all of the optimization problems so far, but each has a well-known graphical interpretation [3,5] in respect of the aerodynamic glide polar (figure 4*a*). For example, the best glide speed (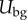) corresponds to the point at which a straight line drawn through the origin is tangent to the curve, because this is where the ratio of aerodynamic sink rate (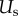) to airspeed (*U*) is minimized (figure 4*a*). Less obviously, because the bird's ground velocity is the sum of its air velocity and the local wind velocity, the airspeed at which the cost of transport is minimized in an updraft (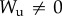) or a headwind (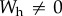) can be found by drawing the line tangent to the curve from an origin displaced downward by 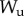 and rightward by 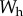 (figure 4*a*). Unfortunately, although this mode of presentation is convenient for identifying theoretical optima, it does not lend itself to displaying multiple solutions simultaneously, because every point (*U*, 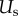) on the aerodynamic glide polar is optimal for multiple combinations of updraft and headwind (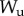, 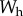). Furthermore, whereas airspeed (*U*) can be measured using a pitot tube, there is no straightforward way to measure the aerodynamic sink rate (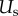) using onboard instrumentation, because barometric or GPS measurements of altitude only provide information on the inertial sink rate (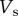). Both limitations can be overcome by plotting the inertial glide polar instead of the aerodynamic glide polar (figure 4*b*). This has two key advantages. First, it allows us to unpack the glide polar by updraft speed, so that every point (*U*, 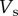) on the axes corresponds to a unique optimum for a specific combination of updraft and headwind (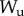, 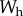), allowing multiple solutions to be displayed simultaneously. Second, it allows empirical data on airspeed (*U*) and inertial sink rate (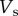) to be plotted directly on the same axes.

Seen in this light, any combination of airspeed and inertial sink rate (*U*, 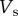) may be optimal for some unique combination of headwind and updraft speed (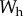, 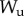). Thus, if we treat every point (*U*, 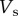) on the axes of figure 4*b* as representing an optimum at which the aerodynamic cost of transport is minimized under some unique combination of updraft and headwind (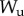, 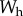), then the inertial glide polar for a given updraft speed (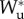) represents the line of optima for 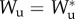. Likewise, the locus of points at which the aerodynamic cost of transport is minimized for a given headwind speed (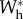) represents the line of optima for 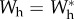 (figure 4*c*). The meaning of this is best understood with reference to the resulting glide optimization chart (figure 4*d*): for any given combination of headwind and updraft speed (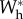, 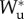), the optimum combination of airspeed and inertial sink rate is found at the intersection of the lines of optima for 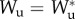 and 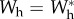. In cases where the updraft strength exceeds the aerodynamic sink rate, the bird will be climbing (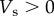) and therefore gaining mechanical energy at a rate 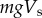 while still making progress over the ground. In such cases, it no longer makes sense to assume that the cost of transport will be minimized (see also §2b), so it is not possible to make any unambiguous prediction of optimal airspeed. Specifically, whereas the bird will maximize the rate at which it harvests mechanical energy while climbing by flying at its minimum sink rate, higher flight speeds may still be optimal if there is any advantage associated with horizontal travel.

## 3. Empirical analysis of flight in a moving atmosphere

The theoretical analysis has been kept as general as possible to this point, but we now change tack to provide as specific a test of the theory as possible, using empirical data collected from a captive-bred male steppe eagle *A. nipalensis* (see table 1 for morphological measurements) soaring freely over windward ridges in the Black Mountains, Wales, UK over the course of 45 separate flights. The bird was released at one of several sites chosen according to wind direction on the day of the test, and was left to fly freely until the end of the flight test, when it was called back by its handler to feed. Individual flight tests were of variable duration, and were set up so as to encourage the bird to loiter over a windward ridge. It is difficult to predict what specific aspects of its flight performance the bird might have sought to optimize under such conditions, but the resulting pattern of flight was typical of what many soaring birds can be observed doing on a daily basis—soaring back and forth along a ridge, and making opportunistic use of thermals to gain altitude.

### (a) Methods

The dataset that we analyse here was described previously in reference [1], so we provide only a brief summary of the experimental methods here. An instrumentation package weighing less than 0.075 kg with battery (*ca* 3% body mass) was worn dorsally on a removable falconry harness (Marshall Direct Ltd, Lancashire, UK). We used an ArduPilotMega2 board (3D Robotics Inc., San Diego, CA) running customized software [31], comprising an MPU6000 IMU measuring three-axis angular velocity, acceleration and Earth magnetic field data at 50 Hz (InvenSense Inc., Sunnyvale, CA), an MT3329 GPS unit estimating position and groundspeed at 10 Hz (MediaTek Inc., Hsinchu City, Taiwan), an MS5011 barometer measuring atmospheric pressure and hence altitude at 10 Hz (Measurement Specialities Inc., Hampton, VA) and an MPXV7002DP differential pressure sensor attached to a pitot tube measuring dynamic pressure and hence airspeed at 10 Hz (NXP Semiconductors Netherlands B.V., Eindhoven). We post-processed the IMU outputs using an extended Kalman filter to estimate heading, pitch attitude and bank angle [31].

Here, we consider only the subset of data corresponding to straight equilibrium gliding flight, for which the estimated bank angle should be close to zero, and the sensed acceleration close to 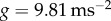. (An accelerometer works by sensing the effective weight of a proof mass, and therefore reads 1 *g* when the inertial acceleration of the device is zero.) Practically speaking, we analysed only those sections of flight for which the estimated bank angle remained 0° ± 5° for at least 3 s, and for which the mean acceleration averaged 1 *g* ± 0.1 *g* over the same interval. This left us with a total of 21 min of data from 36 flights, which we subsampled to give *n* = 420 non-overlapping 3 s intervals of straight gliding flight. For each 3 s interval, we calculated the mean airspeed *U* (0.1 ms^−1^ RMS error at 16.0 ms^−1^; uncorrelated error model), and mean groundspeed *V* (0.1 ms^−1^ RMS error; fully correlated error model). We also calculated the net change in barometric altitude over the same interval, and used this to estimate the mean inertial sink rate (±0.1 ms^−1^ RMS error; uncorrelated error model). In the analyses that follow, we treat these *n* = 420 data points as if they were independent, noting that there is a risk of non-independence for any strictly consecutive data points.

### (b) Adjustment of airspeed in headwinds

For most of the straight glides that we identified, the bird was experiencing a headwind component opposing its air velocity vector. In some cases, this was because it was flying directly into the prevailing wind having climbed away from the ridge in a thermal, and in this case, we would expect there to have been no significant updraft or sidewind component. This being so, we would expect the bird to have flown at an airspeed 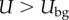 on these straight glide sections, if it were behaving so as to minimize its aerodynamic cost of transport (see §2b). In other cases, the bird was flying a straight track along the ridge, approximately perpendicular to the prevailing wind, and was therefore flying in a pure crosswind relative to its ground velocity vector. In this case, the bird would again have been experiencing a headwind component opposing its air velocity vector, but would also have been experiencing a sidewind perpendicular to its air velocity vector, as well as the updraft associated with the ridge. In this case, the predictions of §2b are ambiguous because whereas the combination of headwind and sidewind components in a crosswind will always have the net effect of increasing the bird's cost of transport, the effect of an updraft will always be to reduce it. In reality, the airspeeds recorded during these glides were all in the range 10.4–26.3 ms^−1^, averaging 16.0 ± 2.9 ms^−1^ (mean ± s.d.), which is considerably faster than the best glide speed of *U*_bg_ = 11 ms^−1^ predicted by all three of the aerodynamic models, even accounting for the additional mass of the instrumentation carried by the bird. It is fortuitous that the three models agree in their predictions for this species (figure 3), but our finding that the bird flew faster than its best glide speed under unfavourable wind conditions is therefore robust to the model uncertainty discussed in §2d.

**Figure 5.**
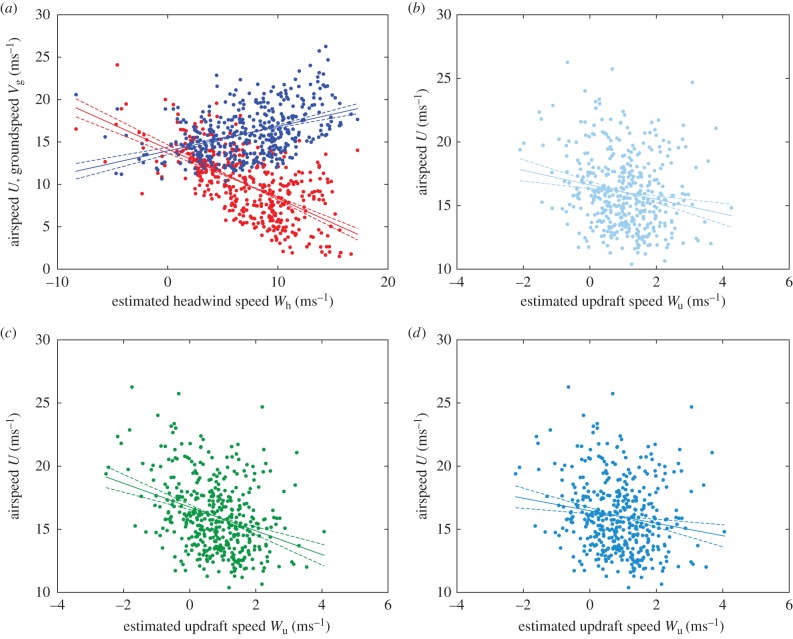
Flight speed versus wind components in a captive steppe eagle (*Aquila nipalensis*) during straight and interthermal glides. Individual data points represent the mean over 3 s. Dashed lines denote the 95% CI on the slope of the regression line, pivoted about the mean for all *n* = 420 data points. (*a*) Airspeed (blue) and groundspeed (red) versus estimated headwind speed. (*b*–*d*) Airspeed versus estimated updraft speed under each of the three aerodynamic models of the glide polar at optimal span (equation (2.12)): light blue [39], green [37] and dark blue [17].

It is possible to go further than this, by combining the inertial data and airspeed measurements to estimate horizontal wind velocity [31]. We used the GPS data to estimate the bird's track angle and groundspeed, which together gave us an estimate of the bird's ground velocity. We then used the yaw angle estimate from the extended Kalman filter to determine the bird's heading, and used the airspeed measurement from the pitot tube to estimate the bird's airspeed, which together gave us an estimate of the bird's air velocity, assuming zero sideslip. Finally, we calculated the vectorial difference between the bird's ground and air velocities, so as to provide an estimate of the horizontal wind velocity, and used this together with our knowledge of the bird's heading to determine the headwind component opposite to the bird's air velocity vector. We then used the resulting data to test how airspeed (*U*) and groundspeed (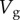) varied with headwind speed (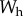). Although the relationships are noisy (figure 5*a*), groundspeed decreased with increasing headwind speed (linear regression: 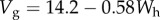; 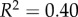, 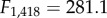, 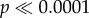), whereas airspeed increased with increasing headwind speed (linear regression: 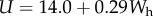; 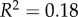, 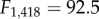, 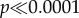). These regressions fitted to onboard data from a captive steppe eagle during straight and interthermal glides are remarkably similar to the regressions fitted by Spaar & Bruderer [21] to radar data from migrating steppe eagles during interthermal glides (groundspeed: 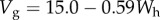; 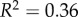, 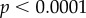; airspeed: 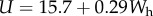, 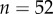, 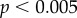). In particular, the slopes of the corresponding relationships are practically identical, which gives us confidence in both datasets, demonstrating a remarkably high degree of reproducibility.

These relationships are qualitatively consistent with what we would expect to see if steppe eagles adjust their airspeed in response to headwinds so as to minimize their aerodynamic cost of transport during straight and interthermal glides. In the absence of such a response, we would have expected to see no relationship between airspeed and headwind, and would have expected groundspeed to decrease linearly with headwind with a slope of minus one. As it is, the attenuated slope for groundspeed and the existence of a positive relationship for airspeed are both consistent with the prediction that birds should fly at a higher airspeed when the headwind speed is higher (§2b). This in turn implies that steppe eagles are capable of estimating headwind speed—presumably by fusing visual information on groundspeed with aerodynamic information on airspeed.

### (c) Adjustment of airspeed in updrafts

Having shown that our steppe eagle increased its airspeed in response to headwinds, we now ask whether there is any evidence that it adjusted its airspeed in relation to updraft speed during straight glides. This is harder to test empirically, because there is no direct way to measure updraft speed using onboard instrumentation. Instead, we are left having to estimate updraft speed from measurements of airspeed and inertial sink rate by solving the inertial glide polar for the unknown updraft (equation (2.13)). The resulting estimates of updraft speed are only as good as our aerodynamic model, so given the uncertainty surrounding the numerical coefficients (§2d), it is important that we bracket our estimates by estimating the updraft speed separately under each of the different models. Figure 5*b–d* therefore plots airspeed against estimated updraft strength for each of the three aerodynamic models [17,37,39] assuming that span is adjusted optimally (equation (2.12)). The fitted regression relationships are in the expected direction, showing a decrease in airspeed with increasing updraft strength, but show considerable variation in slope between the different aerodynamic models, and are too noisy to be taken very seriously (figure 5*b* [39]: 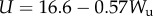; 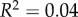, 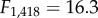, 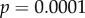; figure 5*c* [37]: 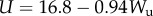; 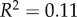, 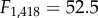, 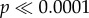; figure 5*d* [17]: 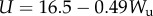; 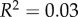, 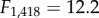, 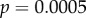). The regressions for the maximal-span case are qualitatively similar, but are only statistically significant for two of the three aerodynamic models (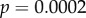, [37]; 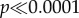, [17]). In any case, it is clear that the very high levels of stochastic noise in these relationships cannot be attributed to the systematic uncertainty in the underlying aerodynamic models. Hence, given that the variation apparent in figure 5 is an order of magnitude greater than the measurement error for the various sensors (§3a), we conclude that our steppe eagle did not consistently adjust its airspeed and/or wing span in relation to updraft strength during straight and interthermal glides. There is therefore no strong evidence in our data to suggest that steppe eagles are capable of estimating updraft strength.

### (d) Adjustment of airspeed in combined headwinds and updrafts

It is possible, in principle, that some of the noise in figure 5 might be attributable to the fact that airspeed is expected to be optimized jointly, rather than separately, in respect of updraft and headwind strength. This can be crudely tested by regressing airspeed on headwind and updraft speed together, which results in models with rather higher predictive power (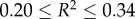 for the three aerodynamic models [17,37,39] at maximal and optimal span). This approach offers some evidence that the airspeed is optimized jointly in respect of headwind and updraft speed, because the variation explained by updraft speed having first controlled for headwind is greater than the variation explained by updraft speed alone, and vice versa. However, a better approach, given the underlying nonlinearities, is to make use of the glide optimization chart that we developed earlier (figure 4*d*).

**Figure 6.**
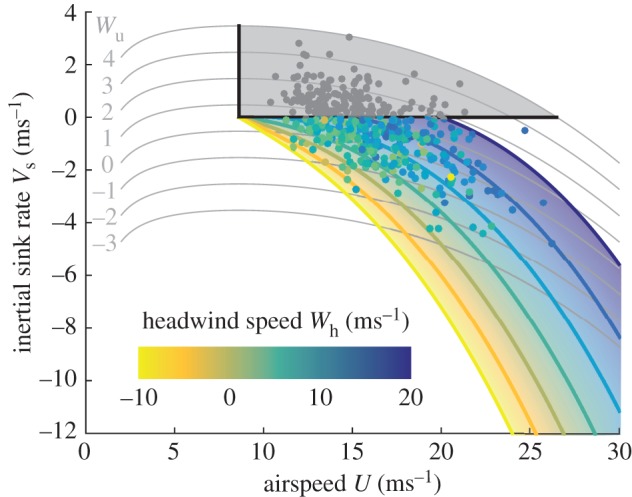
Measured airspeed and inertial sink rate of a captive steppe eagle *Aquila nipalensis* during straight and interthermal glides, plotted on the glide optimization chart (figure 4) for a representative aerodynamic model at maximal span [17]. Individual data points represent the mean over 3 s for *n* = 420 non-overlapping intervals. Estimated headwind speed is shown by the colour of the plotted points; expected headwind speed is indicated by the semi-transparent background shading. If the bird were optimizing its airspeed so as to minimize its aerodynamic cost of transport, then the colours of the plotted points should match the background hue. The background and points are plotted in grey over the region of the chart for which the bird is climbing, where the optimization breaks down.

If the bird were adjusting its airspeed so as to minimize its aerodynamic cost of transport, then every measured combination of airspeed and inertial sink rate would be expected to be associated with some unique combination of headwind and updraft, neglecting the effects of sidewinds. This expected combination of headwind and updraft speed can be read off the glide optimization chart directly, or can be solved for analytically. Either way, the result is an expected headwind that can be compared with the estimated headwind that was obtained previously from the bird's air and ground velocities. By way of illustration, figure 6 presents this comparison for a representative aerodynamic model [17] shown here at fixed-span, plotting each measured combination of airspeed and inertial sink rate on the glide optimization chart. The chart itself is coloured by the expected headwind, whereas the data points are coloured by the estimated headwind. Hence, if the bird were adjusting its airspeed so as to minimize its cost of transport as predicted by the model, then the colours of the plotted points would match the background hue. It is clear by inspection of figure 6 that the measured and expected headwinds are broadly similar, but there are some obvious outliers.

**Figure 7.**
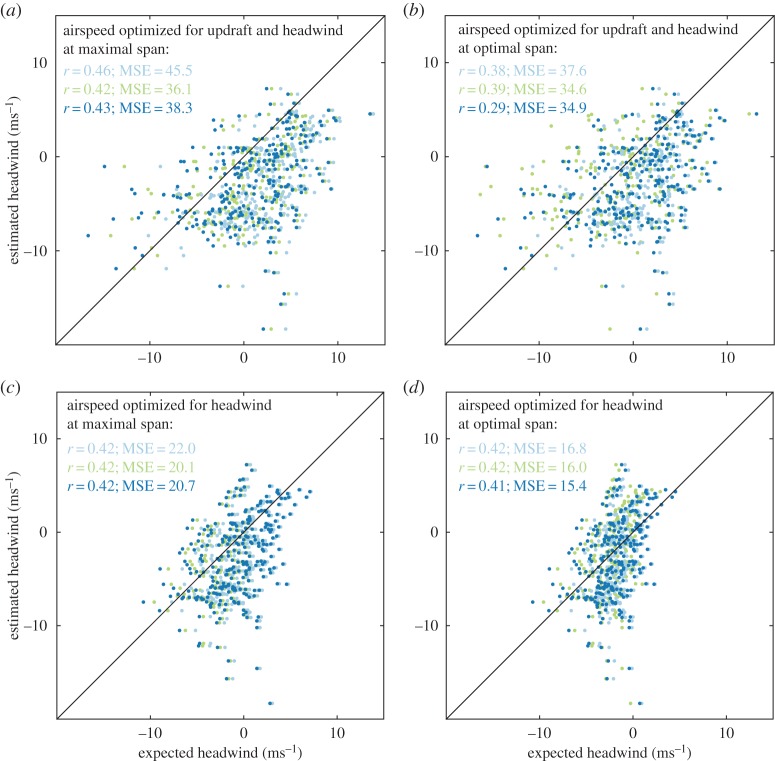
Estimated versus expected headwind for a captive steppe eagle *Aquila nipalensis* under each of the three published aerodynamic models: light blue [39], green [37] and dark blue [17]. The expected headwind is predicted under four different sets of assumptions (see panel titles), optimizing airspeed so as to minimize the cost of transport in relation to headwind and updraft speed (*a,b*), and in relation to headwind speed only (*c,d*), at either optimal or maximal span. Black line, identity line; *r*, linear correlation coefficient; MSE, mean-squared error between estimated and expected headwinds.

Figure 7 plots the estimated versus expected headwind for all three aerodynamic models, excluding any points for which the bird was climbing. (The aerodynamic cost of transport is negative in this case, so may no longer make sense as a minimand.) Interestingly, the measured and expected headwinds are positively correlated (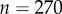, 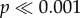) for all of the aerodynamic models at both maximal (figure 7*a*) and optimal (figure 7*b*) span. However, there is an obvious systematic bias in the predictions, because the estimated headwind is typically higher than the expected headwind (figure 7*a,b*). This is manifest in the high mean-squared error between the estimated and expected headwinds. For comparison, figure 7*c,d*, therefore, plots estimated versus expected headwind assuming that the bird takes no account of updraft speed—instead behaving as if it were flying in only a horizontal headwind. In this case, the measured and expected headwinds remain positively correlated (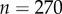, 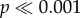), but the bias in the predictions is largely removed, and the mean-squared error is greatly reduced. It follows that the flight data are better explained by assuming that the bird took no account of the local updraft speed, consistent with the conclusion of §3c.

### (e) Summary

In summary, there is strong evidence that our steppe eagle adjusted its airspeed in relation to headwind speed during straight and interthermal glides, and that it did so in a manner that matches quantitatively what we would expect to see if it were attempting to minimize its cost of transport. In contrast, although there is some evidence that our steppe eagle adjusted its airspeed in relation to updraft speed, there is no evidence that it did so in a manner that would be expected to minimize the cost of transport.

## 4. Conclusion

We have shown here how empirical data obtained from onboard instrumentation can be used to test and develop the underlying theory of soaring and gliding flight. We have shown in particular that our captive-bred steppe eagle adjusted its airspeed with respect to headwind speed in precisely the way that the theory predicts, if the aerodynamic cost of transport were being minimized. Moreover, the slopes of the fitted relationships are practically identical to those fitted to radar data from migrating steppe eagles during interthermal glides [21]. These relationships are similar to those found in other birds [20], in that groundspeed typically decreases (increases) linearly with headwind (tailwind) speed with a gradient less than one (see also [6]). In contrast, we have found only weak evidence that our steppe eagle adjusts its airspeed with respect to updraft speed. This finding is somewhat at odds with the radar data from migrating steppe eagles, which do appear to show a reduction in airspeed when gliding in a straight line in an updraft [21], but it is possible that updraft speed is simply a difficult quantity for a bird to estimate and respond to appropriately.

Perhaps the greatest limitation of the empirical approach that we have used here is the lack of information on how wing span is adjusted in flight, coupled with the fact that the glide polar model (equation (2.13)) takes no account of the effects of variable tail span (but see [47]). Obtaining continuous measurements of wing and tail span in the field presents a difficult, but not insurmountable technical challenge, which future work will have to overcome. Nevertheless, variable span has a smaller effect on the glide polar than might otherwise be guessed, especially at lower airspeeds (figure 3), and most of the conclusions that we have drawn are robust to the uncertainty in wing span. This rather begs the question of *why* birds should reduce wing span with airspeed as they do. We agree with Pennycuick's general conclusion that this probably has some advantage to do with speed control rather than glide optimization [37], and propose that this advantage relates to keeping the wings at a reasonable angle of attack across flight speeds. Specifically, in order to move along the glide polar at fixed span, a bird must decrease its lift coefficient, and hence its angle of attack, with increasing airspeed. Birds' wings are highly twisted, so too great a reduction in the overall angle of attack would be expected to lead to negative loads being taken on some parts of the wing—particularly when flying through turbulence [1]. We therefore hypothesize that birds reduce span with increasing airspeed to attenuate the reduction in angle of attack that would otherwise occur. This would lead naturally to a control paradigm in which span served as the primary speed control, with tail-mediated changes in angle of attack being used to move along the glide polar at a given span (see also [47]).

Finally, we note that there is considerable scope to extend the approaches that we have explored here. In particular, the general theory of the energetics of soaring flight that we have developed (equation (2.4)) is ready to use in assessing the rate of energy harvesting through soaring under theoretical or field conditions, and allows quantitative predictions to be made about how birds should respond to different patterns of variation in wind conditions over space and time (see also equation (2.7)). For example, given that turbulent air offers the possibility of gust soaring, we might expect soaring birds to seek out regions of turbulent air that they can exploit for energy harvesting. Likewise, the simple observation that the induced drag equals the combined parasite and profile drag at best glide speed (equation (2.10)) offers an easy way to estimate both the induced drag factor (*k*) and the equivalent flat plate area for the combined parasite and profile drag, if inertial sink rate and airspeed can be measured using onboard instrumentation in a maximally shallow glide through still air. The prospects for future research in this area are bright.

## Data accessibility

The raw mean data for each of the *n* = 420 non-overlapping 3 s intervals of straight gliding flight are appended as a text file in the electronic supplementary material.
